# Exercise preconditioning alleviates photothrombotic ischemic stroke in mice by orchestrating neutrophils

**DOI:** 10.3389/fphys.2025.1580283

**Published:** 2025-07-04

**Authors:** Tae Yeon Kim, Yun Seo Cho, Jae Yeon Park, Songwon Woo, Kihoon Yuk, Dong Heon Yi, In cheol Jeong, Jin Pyeong Jeon, Yoe-Sik Bae, Min-Chul Lee, Hyo Youl Moon

**Affiliations:** ^1^ Department of Physical Education, Seoul National University, Seoul, Republic of Korea; ^2^ School of Artificial Intelligence Convergence, Hallym University, Chuncheon, Republic of Korea; ^3^ Department of Population Health Science and Policy, Icahn School of Medicine at Mount Sinai, New York, NY, United States; ^4^ Department of Neurosurgery, Hallym University College of Medicine, Chuncheon, Republic of Korea; ^5^ Department of Biological Sciences, Sungkyunkwan University, Suwon, Republic of Korea; ^6^ Department of Sports Medicine, College of Health Science, CHA University, Pocheon, Republic of Korea; ^7^ Learning Sciences Research Institute, Seoul National University, Seoul, Republic of Korea; ^8^ Institute of Aging, Seoul National University, Seoul, Republic of Korea

**Keywords:** exercise preconditioning, neutrophil extracellular traps, migration capacity, photothrombotic ischemic stroke, voluntary exercise

## Abstract

**Background:**

In ischemic stroke (IS) pathology, neutrophils are rapidly recruited to the infarcted brain and exacerbate tissue damage by releasing the amount of neutrophil extracellular traps (NETs). Previous studies have demonstrated that alleviating IS is associated with reduced accumulated neutrophils and NET levels. Notably, exercise preconditioning (EP) has the potential to modulate neutrophil function, thereby contributing to the amelioration of IS. This study compared the functional differences between resting and EP-induced neutrophils under IS pathology.

**Methods:**

Eight-week-old C57BL/6J male mice underwent 8 weeks of voluntary exercise before photothrombotic ischemic stroke (PTI) surgery. Behavioral tests were conducted 2 days after PTI to verify the effects of EP on acute recovery following PTI. To evaluate whether EP affected neutrophil recruitment and chemotactic signaling, RT-qPCR was performed on infarcted cortical regions. A migration assay was performed to assess the migration capacity of blood-isolated neutrophils under IS. Plasma NET levels were quantified using enzyme-linked immunosorbent assay (ELISA), while NET presence in the brain was evaluated through immunofluorescence (IF) staining.

**Results:**

Exercise-preconditioned-PTI (EP-PTI) mice showed superior behavioral outcomes in grip strength and open-field tests both before and 2 days after PTI compared to sedentary-PTI (Sed-PTI) mice. EP also suppressed the expression of chemotactic signaling molecules following PTI, suggesting reduced inflammatory recruitment and infiltration. Furthermore, EP reduced the migration capacity of neutrophils and decreased NET formations in both plasma and brain 2 days after PTI surgery.

**Conclusion:**

Our study demonstrates that EP enhances acute recovery in IS and may beneficially orchestrate IS pathology by inhibiting the migratory capacity and reducing NET formation *in vivo*.

## 1 Introduction

Stroke is an emergent brain disease that causes severe disability and mortality (Feigin et al., 2022). Ischemic stroke (IS) leads to brain damage and impairments in motor functions that are crucial for a healthy life (Ivey et al., 2005; Rudberg et al., 2021). Previous studies have emphasized the importance of advanced prevention and acute recovery, which have been shown to lower mortality in patients with IS (Benjamin et al., 2017). As a result, there is an increased demand for effective prevention strategies under IS.

Exercise preconditioning (EP) is considered one of the effective prevention strategies (Otsuka et al., 2016; Islam et al., 2017; Hafez et al., 2021) that alleviate the symptoms or conditions of IS by reducing infarct volume, brain edema, inflammation signaling, and neurological dysfunction (Otsuka et al., 2016; Islam et al., 2017; Wang et al., 2020; Hafez et al., 2021). Additionally, exercise has the potential to modulate both the quantity (Pedersen and Hoffman-Goetz, 2000) and function of neutrophils under IS pathology (Gavrieli et al., 2008; Ondracek et al., 2022).

Neutrophils are the key therapeutic targets for IS, as they worsen the disease condition during the early stages (Jickling et al., 2015; Cai et al., 2020; Zhao et al., 2023). After IS, neutrophils accumulate in the brain and become excessively activated. For example, neutrophil extracellular traps (NETs), which trap and kill pathogens while regulating inflammation, are excessively formatted or dysregulated, causing various inflammatory responses and acting as a major trigger for thrombosis. Stroke symptoms can be alleviated by reducing the number of neutrophils at infarct sites (Price et al., 2004; Perez-de-Puig et al., 2015; Kang et al., 2020) and suppressing the activities of neutrophils, such as NETs (Vallés et al., 2017; Denorme et al., 2022).

Exercise can potentially modify the recruitment capacity of neutrophils (Gavrieli et al., 2008; Borges et al., 2018) and decrease the formation of NETs (Yazdani et al., 2021; Ondracek et al., 2022). However, there has been no research on the effects of EP on neutrophils in IS pathology. This research focuses on demonstrating evidence of the effects of EP on neutrophils in a photothrombotic ischemic stroke (PTI) mouse model. Our main hypothesis was that EP would alter the migration capacity of neutrophils and change the NET levels in PTI conditions.

## 2 Methods and materials

### 2.1 Mice

Male C57BL/6J mice (8 weeks old, 21–25 g, DBL, Eumseong, Republic of Korea) were used in this study. We randomly divided the mice into three groups: Control (Con, n = 8), Sedentary-PTI (Sed-PTI, n = 10), and Exercise-preconditioned-PTI (EP-PTI, n = 10). Each mouse was housed individually and weighed weekly using a laboratory balance.

### 2.2 Voluntary exercise protocol

The exercise precondition group was assigned to the voluntary wheel cage (radius of 75.36 cm), which was connected to a wheel counter to monitor running activity continuously for 8 weeks (Tecniplast, West Chester, United States). For habituation, mice were accustomed to a running wheel for a week.

### 2.3 Photothrombotic ischemic stroke (PTI) surgery

Under isoflurane anesthesia (2% in an oxygen/air mixture), the mice were positioned in a stereotactic frame (Jeong Do Bio & Planet Co., Seoul, Republic of Korea). We then injected Rose Bengal (Sigma, St. Louis, United States) at a dose of 3 μL/g (10 mg/mL, dissolved in 0.9% saline) into a retro-orbital injection. After 5 min, the skull was exposed, and a cold light source (step 34, 184–204 lm, Carl Zeiss, Oberkochen, Germany) was directed onto the skull under isoflurane anesthesia (3.5% in an oxygen/air mixture) (Toda et al., 2014). The cold light beam was directed from 2.5 mm anterior to 0.5 mm posterior and 0–4.0 mm lateral to the bregma to induce an infarct site in the left motor cortex for 15 min. Following the completion of the surgery, the scalp was clamped and sterilized. The survival rate was 100% in the Sed-PTI and 90% in the EP-PTI group.

### 2.4 Grip strength

We used a grip strength meter (Bioseb, Vitrolles, France) (Balkaya et al., 2013; Ruan and Yao, 2020). The test was repeated three times with 5-min breaks between trials, and maximum force was recorded in grams (g). Grip strength data were normalized to the weight of mice and calculated as an average of three trials.

### 2.5 Open field test

We measured the total distance and velocity over 5 min to evaluate the locomotor and general activity levels (Balkaya et al., 2013; Rezaei et al., 2017; Camargos et al., 2020; Ruan and Yao, 2020). Velocity was calculated by dividing the distance (m) covered by the time mobile (sec). The test was conducted using a box (40 cm × 40 cm × 40 cm) equipped with a video tracking camera. During the test, the mice were placed in separate spaces with no people nearby. Data were analyzed using ANY-Maze software (Stoelting Co., Wood Dale, United States).

### 2.6 Blood neutrophil isolation and migration assay

The neutrophils were isolated from blood using a neutrophil enrichment kit (STEMCELL Technologies, Vancouver, Canada). The detailed steps were performed according to the manufacturer’s instructions.

Migration assay of neutrophils was evaluated using 8 μm pore size, 96-well Transwell plates (CORNING, New York, United States). Blood-isolated neutrophils were resuspended in the media containing 2% FBS in RPMI 1640 (Welgene, Gyeongsan, Republic of Korea) and seeded at 1 × 10^5^ cells in the upper chamber. Next, fMLP (1 μM, Sigma, St. Louis, United States) was loaded into the lower chamber. The plates were then incubated for 2 h at 37°C. The migrated cells were stained with hematoxylin for 5 min and counted in five fields using a grid with a manual counter under a bright field light microscope (Carl Zeiss, Oberkochen, Germany).

### 2.7 Differential quick staining

A Diff-Quik staining kit (Polyscience, Warrington, United States) was used to confirm the presence of neutrophils. The blood-isolated neutrophils were treated with poly-L-lysine (Sigma, St. Louis, United States) for 5 min and loaded with 2 × 10^5^ cells per 8-well chamber slide (Thermo Fisher Scientific, Waltham, United States). The detailed steps were performed according to the manufacturer’s instructions. Images were captured using an optical microscope (Carl Zeiss, Oberkochen, Germany).

### 2.8 Quantitative real-time PCR (qRT-PCR)

Total RNA was isolated from the left infarcted cortex 2 days after PTI surgery using TRIzol reagent (Sigma, MO, United States), according to the manufacturer’s protocol. cDNA was synthesized from 1,000 ng of total RNA using Accupower® CycleScript RT PreMix (Bioneer, Daejeon, Korea). Quantitative PCR was performed to evaluate the relative expression levels of Ly6G, fMLP, CXCR2, Zonula Occludens-1 (ZO-1), and occludin. The sequences of primers were as follows: Ly6G 5′-TGCCCCACTACTCTGGACAA-3′ and 3′-AGGACTGAAACCAGGCTGAA-5’; fMLP 5′-ATTGCACTGGACCGCTGTAT-3′ and 3′-TCCAGGGGGAGAAGTCGAAA-5’; CXCR2 5′-GGGTCGTACTGCGTATCCTG-3′ and 3′-AGACAAGGACGACAGCGAAG-5’; ZO-1 5′- GGGAGGGTCAAATGAAGACA-3′ and 3′-GGCATTCCTGCTGGTTACAT-5’; GAPDH 5′-AAGGTCGGTGTGAACGGA-3′and 3′-GATGGGCTTCCCGTTGATGA-5’.

Each reaction was prepared by mixing 2 μL of cDNA with 18 μL of PreMix (2 μL primer mix, 7 μL nuclease-free water, and 10 μL SYBR Green) for a total volume of 20 μL per well. All reactions were carried out in duplicate. Gene expression levels were normalized to the reference gene *GAPDH*, and relative quantification was performed using the 2^−ΔΔCT^ method. Data are presented as relative fold changes compared to the control group. The PCR protocol was initiated with a denaturation step at 95°C for 2 min, followed by 40 cycles of amplification consisting of denaturation at 95°C for 5 s, annealing at 56°C for 10 s, and extension at 72°C for 15 s. Fluorescence data were collected at the end of each cycle. A melting curve was generated by progressively increasing the temperatures from 70°C to 95°C in 0.5°C increments.

### 2.9 Enzyme-linked immunosorbent assay (ELISA)

We centrifuged whole blood (1,000 ×g, 10 min) and collected plasma. The following ELISA kits were used: S100 calcium-binding protein B (S100B) (1:50, Elabscience, Wuhan, China), N-terminal pro-b-type natriuretic peptide (NT-pro BNP) (1:5, Elabscience, Wuhan, China), and citrullinated histone H3 (CitH3) (1:5, Cayman, Ann Arbor, United States). We followed detailed steps according to the manufacturer’s instructions with modifications to the incubation times. Specifically, incubation times were 15 min for S100B, 20 min for NT-pro BNP, and 1 h for CitH3. The plates were analyzed using a NanoQuant Infinite M200 spectrophotometer (TECAN, Mannedorf, Switzerland).

### 2.10 Histology

We perfused mice with 20 mL of 1X PBS, followed by 15 mL of 4% PFA, and then isolated the whole brain tissue. Isolated brains were fixed in 4% PFA and dehydrated with 15% and 30% sucrose in distilled water, with each step carried out for 24 h. Subsequently, brains were embedded in the Tissue-Tek optimum cutting temperature (O.C.T) compound (Sakura Finetek, Tokyo, Japan). We sectioned the tissue directed from 3 mm anterior to 1 mm posterior to the bregma with 24 μm thickness for Nissl staining and 14 μm thickness for IF staining using a cryostat microtome (BKKD-3000, China) and stored it in a −80°C deep freezer.

#### 2.10.1 Nissl staining

Nissl staining was used to visualize the PTI-induced infarct site in the brain. Brain sections were dehydrated in serially diluted ethanol, starting at 70% and progressing to 95%. Following this, brain sections were incubated in a 0.1% cresyl violet solution (Thermo Fisher Scientific, Waltham, United States) for 5 min at 37°C. Sections were dehydrated in an ascending alcohol series and washed with xylene. The images were captured using a scanner (3DHISTECH, Budapest, Hungary).

#### 2.10.2 Immunofluorescence (IF) staining

Before use, the brain sections were fixed in 4% PFA for 15 min and then blocked with a blocking buffer composed of 5% animal serum with 0.4% Triton X-100 in PBS (PBS-T) for 1 h. The primary antibodies were incubated overnight at 4°C: glial fibrillary acidic protein (GFAP) (1:500, Abcam, Cambridge, England) and CitH3 (1:200, Abcam, Cambridge, England). The following day, the brain sections were washed with PBS and incubated with Alexa Fluor 555-conjugated goat anti-rabbit secondary antibodies (1:500, Invitrogen, Carlsbad, United States) for 1 h 30 min. Samples were washed with PBS and then incubated with DAPI (0.1 μg/mL, Sigma, St. Louis, United States) for 5 min. For GFAP staining, images were obtained from the hippocampus, subgranular zone (SGZ), subcallosal zone (SCZ), and corpus callosum. In contrast, CitH3 staining was conducted on the ischemic cortex (infarct core) and its surrounding penumbra region. Additionally, we captured 8 to 12 fields of view for both markers, and the average of these fields was used for the analysis.

### 2.11 Statistical analysis

Statistical analyses were performed using GraphPad Prism software 10.1.2 (GraphPad Software Inc., San Diego, United States). Depending on the experimental design, data were analyzed using Student’s t-test, one-way ANOVA, or two-way ANOVA, followed by Bonferroni *post hoc* tests. Specifically, Student’s t-test was used for comparisons between two groups (Sed-PTI versus EP-PTI), in the migration assay and the quantification of CitH3-positive cells in the ischemic brain. One-way ANOVA was applied to compare three groups in ELISA and PCR. To evaluate the effects of EP and PTI surgery on physiological and motor function, we conducted a two-way ANOVA with group (Sed and EP) and time (before and after PTI) as factors for grip strength, total distance, and velocity. The data were given as mean ± standard deviation. p-values <0.05 were considered statistically significant.

## 3 Result

### 3.1 Exercise preconditioning contributed to improved acute recovery following PTI

**FIGURE 1:**
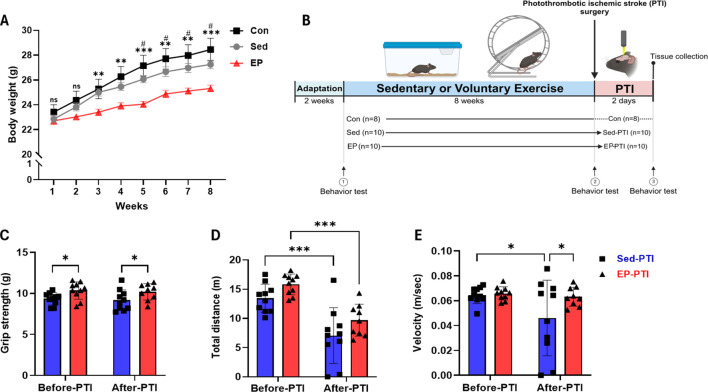
Exercise preconditioning showed notable improvement in grip strength and general activity 2 days after the PTI. **(A)** Mouse body weight monitoring over 8 weeks. Con (n = 8), Sed (n = 10), and EP (n = 10). #*p* < 0.05, ns = not significant, significant differences between Con and EP. *p < 0.05, **p < 0.01, ns = not significant, significant differences between Sed and EP. **(B)** All experiments followed the schematic timeline shown here. Created with BioRender.com (BioRender, Toronto, Canada). **(C)** Grip strength, **(D)** total distance and **(E)** velocity were conducted with Sed-PTI (n = 10) and EP-PTI (n = 9). Data were presented as mean ± SD (*p < 0.05, **p < 0.01, ***p < 0.001). Con: Control; EP-PTI: Exercised preconditioning-photothrombotic ischemic stroke; Sed-PTI: Sedentary-photothrombotic ischemic stroke.

To monitor general health and assess physiological responses to exercise, body weights were measured weekly throughout the 8 weeks (Figure 1A). Body weight showed a significant main effect of group (F(3,70) = 251.1, p < 0.0001) and time (F(2,25) = 6.443, p = 0.0055). Post hoc tests indicated that EP mice exhibited a significantly lower weight gain compared to the Con and Sed mice. Notably, no mice showed an abnormal or outlier-level reduction in body weight during the study period. EP mice exhibited an average weekly running distance of 6.42 ± 0.98 km during the exercise period. The average running distance per week for each mouse is provided in Supplementary Table S1. Subsequently, we conducted PTI surgery after 8 weeks of sedentary or voluntary exercise and performed behavioral tests at each time point (Figure 1B).

Physical exercise decreases motor dysfunctions caused by IS by enhancing muscle strength and general activity, including walking speed (Saunders et al., 2020; Hafez et al., 2021). Grip strength showed a significant main effect of group (F(1,18) = 8.903, p = 0.0080), indicating that EP led to an overall improvement. A post-hoc comparison revealed that the EP-PTI maintained significantly higher grip strength compared to the Sed-PTI both before and after PTI (Figure 1C; p = 0.0354, p = 0.0380), suggesting a sustained benefit of EP on muscle strength. In the open field test, total distance revealed significant main effects of group (F(1,35) = 6.155, p = 0.0181) and time (F(1,35) = 38.82, p < 0.0001). Although baseline measurements showed that EP mice traveled a shorter distance compared to Sed mice before the intervention (Supplementary Figure S1), EP led to a significant increase in total distance after 8 weeks (Figure 1D; p = 0.0214), reflecting enhanced general activity. After PTI, however, both groups exhibited significant reductions in distance traveled (Sed-PTI, p = 0.0003; EP-PTI, p = 0.0008). For velocity, there was a trend toward a main effect of time (F(1,35) = 3.956, p = 0.0546). Notably, post-hoc analysis further revealed a significant reduction in the Sed-PTI compared to Sed (Figure 1E; p = 0.0176), whereas the EP-PTI maintained a velocity similar to baseline. This resulted in a significant difference between EP-PTI and Sed-PTI (p = 0.0296), suggesting that EP mitigated the decline in locomotor function following ischemic injury. Furthermore, two mice in the Sed-PTI group were nearly immobile, suggesting substantial physiological impairment. These behavioral findings indicate that EP may enhance acute motor recovery following ischemic injury.

### 3.2 Exercise preconditioning tends to reduce the level of stroke markers

**FIGURE 2:**
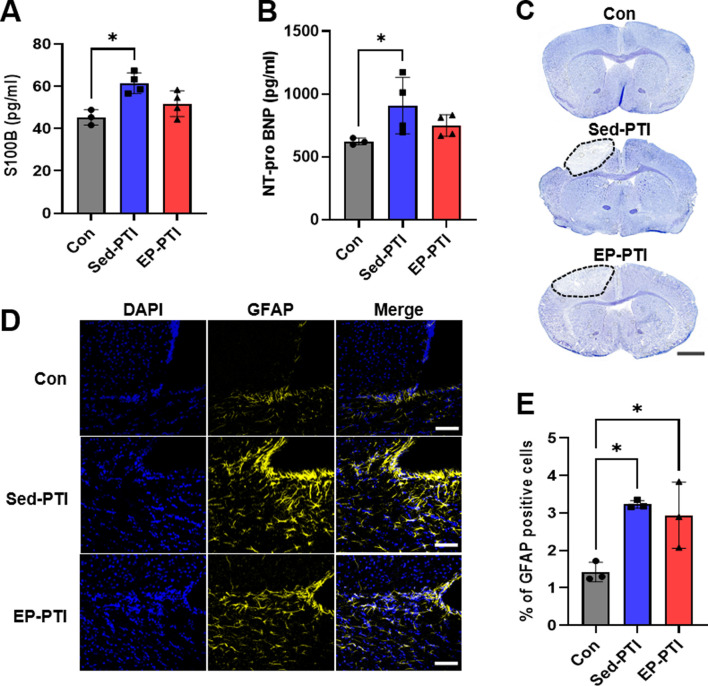
Stroke markers were increased in PTI mice compared to Con. **(A)** ELISA evaluated S100B and **(B)** NT-pro BNP. Con (n = 3), Sed-PTI (n = 4), and EP-PTI (n = 4). **(C)** Photomicrographs depicting Nissl staining of mice brains. Scale bar = 1 mm. **(D)** Representative images of IF staining at a magnification of 20×. Scale bar = 50 µm. **(E)** Percentage of GFAP-positive cells. Con (n = 3), Sed-PTI (n = 3), and EP-PTI (n = 3). Data were presented as mean ± SD (*p < 0.05). Con: Control; EP-PTI: Exercised preconditioning-photothrombotic ischemic stroke; Sed-PTI: Sedentary-photothrombotic ischemic stroke.

S100B, NT-pro BNP, and GFAP are widely recognized biomarkers that increase under IS conditions (Vibha and Misra, 2020; Amalia, 2021). In parallel, the levels of S100B and NT-pro BNP were significantly higher in the Sed-PTI mice than in the Con mice (Figures 2A,B; S100B: p = 0.0281, η^2^ = 0.63; NT-pro BNP: p = 0.0309, η^2^ = 0.59). On the other hand, no significant differences were found between the Control and EP-PTI groups (S100B: p = 0.9234; NT-pro BNP: p = 0.2270) or between the Sed-PTI and EP-PTI groups (S100B: p = 0.2642; NT-pro BNP: p > 0.9999).

Nissl staining was utilized to confirm the location of the infarcted site following PTI, revealing noticeable infarction in the left cortex (Figure 2C). Additionally, IF staining was performed to assess GFAP expression levels in the brain after PTI (Figure 2D). Consequently, GFAP levels were significantly higher in the PTI groups, including Sed-PTI (p = 0.0143) and EP-PTI (p = 0.0306), than in the Con group (Figure 2E; η^2^ = 0.77).

### 3.3 Exercise preconditioning suppressed the expression of neutrophil-related chemotactic signaling molecules after IS

Neutrophils are known to increase both after exercise and during IS (Sand et al., 2013; Cai et al., 2020). To estimate the effects of exercise on neutrophil maturation under non-stroke conditions, we first performed a flow cytometry analysis of bone marrow cells from mice following 8 weeks of voluntary wheel running (Supplementary Figure S2A). Although the total number of neutrophils remained unchanged (Supplementary Figure S2B, p = 0.0938), exercise reduced the proportion of mature neutrophils, which are characterized by higher migratory capacity compared to immature neutrophils (Supplementary Figure S2C; p = 0.0469, Supplementary Figure S2D; p = 0.0297). Next, to examine whether these exercise-induced changes were preserved under IS conditions, we executed a blood cell analysis 2 days after we executed a blood cell 241 analysis 2 days after PTI surgery (Supplementary Figure S3). Nonetheless, there was no significant difference in the percentage of myeloid cells between Sed-PTI and EP-PTI groups under IS conditions (p = 0.4321).

**FIGURE 3:**
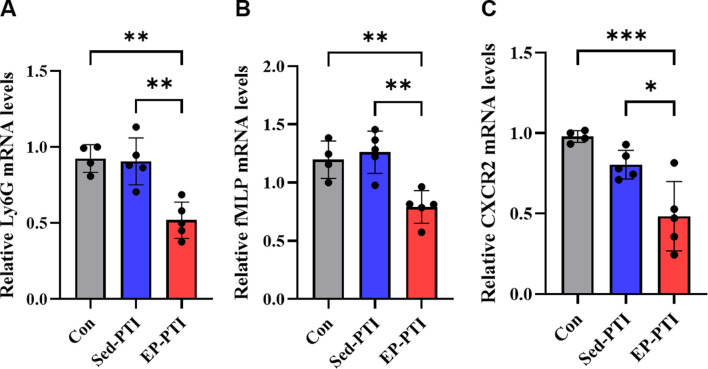
Downregulation of chemotactic signaling molecules was observed only in EP-PTI 2 days after IS. **(A)** Ly6G, **(B)** fMLP, and **(C)** CXCR2 mRNA expression levels in the infarcted region of the brain. Con (n = 4), Sed-PTI (n = 5), and EP-PTI (n = 5). Data were presented as mean ± SD (*p < 0.05, **p < 0.01, ***p < 0.001). Con: Control; EP-PTI: Exercised preconditioning-photothrombotic ischemic stroke; Sed-PTI: Sedentary-photothrombotic ischemic stroke.

To further assess whether EP influenced neutrophil infiltration into the ischemic brain and their chemotactic behavior, RT-qPCR was performed on infarcted cortical regions. Ly6G, a widely used surface marker of neutrophils that reflects the maturation status in mice (Smith and Trinchieri, 2018), was significantly downregulated in the EP-PTI compared to both the Con (p = 0.0018) and Sed-PTI (p = 0.0016) groups (Figure 3A; η^2^ = 0.74), indicating a potential decreases in the infiltration of mature neutrophils into the infarcted region. Additionally, the expression of chemotactic mediators, which are known to enhance neutrophil migration and NET formation, was significantly suppressed by EP. fMLP was markedly decreased in EP-PTI mice compared to Con (p = 0.0097) and Sed-PTI mice (p = 0.0023) (Figure 3B; η^2^ = 0.69). Similarly, CXCR2 was significantly lower in EP-PTI mice than in Con (p = 0.0009) and Sed-PTI (p = 0.0130) mice (Figure 3C; η^2^ = 0.72). Together, these findings suggest that while overall myeloid cell numbers in the blood remain unchanged under stroke conditions, EP modulates neutrophil maturation and suppresses their chemotaxis-related gene expression, thereby potentially reducing excessive neutrophil recruitment to the infarcted brain.

### 3.4 Exercise preconditioning reduce the migration capacity of neutrophils after IS

**FIGURE 4:**
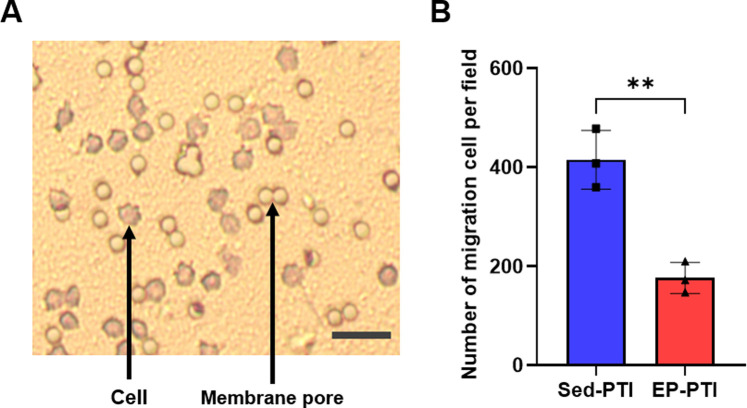
The migration capacity of blood-derived neutrophils was decreased in EP-PTI compared to Sed-PTI mice. **(A)** Photomicrographs of migrating neutrophils at a magnification of 10×. Scale bar = 15 µm. **(B)** Migration assay. Sed-PTI (n = 3) and EP-PTI (n = 3). Data were presented as mean ± SD (**p < 0.01). EP-PTI: Exercised preconditioning-photothrombotic ischemic stroke; Sed-PTI: Sedentary-photothrombotic ischemic stroke.

To assess the migratory capacity, a critical function of neutrophils required for recruitment to the injury site in the initial phase of IS, we isolated neutrophils from blood 2 days after PTI. Following isolation, we conducted Diff-Qick staining to identify the granule morphology of blood-derived neutrophils (Supplementary Figure S4). The migration capacity was then evaluated by counting the number of migrated cells in a Transwell assay (Figure 4A). As a result, neutrophils isolated from EP-PTI mice demonstrated significantly fewer migrated cells per field than those isolated from Sed-PTI mice (Figure 4B; p = 0.0035, Cohen’s d = 6.16). In conclusion, EP can reduce the migratory capacity of neutrophils under IS conditions.

### 3.5 Exercise preconditioning can potentially decrease the NET formation in plasma and brain after IS

**FIGURE 5:**
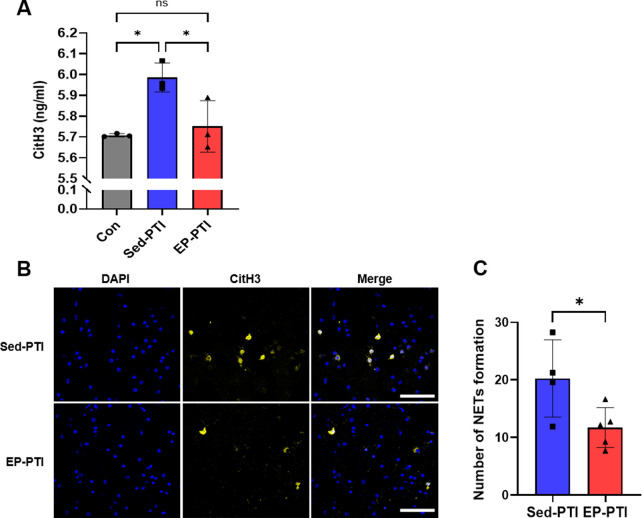
CitH3 was decreased in EP-PTI compared to Sed-PTI mice in both plasma and brain. **(A)** ELISA evaluated CitH3 levels. Con (n = 3), Sed-PTI (n = 3), and EP-PTI (n = 3). **(B)** Representative images of IF staining at a magnification of 40×. Scale bar = 40 µm. **(C)** Number of NETs formations per field. Sed-PTI (n = 4) and EP-PTI (n = 5). Data were presented as mean ± SD (*p < 0.05, **p < 0.01, ns = not significant). CitH3: citrullinated histone H3; Con: Control; EP-PTI: Exercised preconditioning-photothrombotic ischemic stroke; Sed-PTI: Sedentary-photothrombotic ischemic stroke.

NETs are recognized as a major trigger of thrombosis (Vallés et al., 2017; Zhou et al., 2020). To assess NET levels, two separate experiments were conducted to evaluate their formation by circulating and brain-infiltrating neutrophils. Plasma CitH3 levels were significantly elevated in Sed-PTI mice compared to both the Con (p = 0.0184) and EP-PTI (p = 0.0388) groups (Figure 5A; η^2^ = 0.77). On the other hand, EP effectively reduced NET levels to a degree comparable to the Con group (p > 0.9999). Additionally, brain-infiltrating neutrophil-derived NETs were stained, and the number of NETs was quantified (Figure 5B). NET formation in the brain was significantly higher in Sed-PTI mice compared in EP-PTI mice (Figure 5C; p = 0.0417, Cohen’s d = 1.88). These findings indicate that EP reduces NET formation both in the plasma and in the brain.

## 4 Discussion

Stroke is the second leading cause of death worldwide, causing neurological dysfunctions and body weakness (Feigin et al., 2019; Feigin et al., 2022). Advanced treatment strategies hold significant clinical implications for both short- and long-term outcomes in patients. However, due to the limited eligibility of patients for therapy within the timeframe (Saini et al., 2021), the need for strategies aimed at preventing IS has increased (Santos-Gallego et al., 2010). Notably, physical activity is an effective strategy for preventing and managing prognosis outcomes after IS (Saunders et al., 2020; Hafez et al., 2021). In this study, using a PTI stroke mouse model, we demonstrated the impact of EP on the aspect of acute recovery 2 days after induced photothrombotic ischemia.

Exercise is known to enhance motor function and increase general activity in stroke patients, which is highly related to the quality of life after a stroke (Saunders et al., 2014; Martino Cinnera et al., 2020). The first 2 days constitute the acute phase, during which acute recovery is important for favorable prognostic outcomes (Benjamin et al., 2017; Feigin et al., 2022). Our behavioral test results suggest that 8 weeks of EP can enhance acute recovery in the early stage following IS. In particular, exercise-preconditioned mice maintained their walking speed before and after PTI without significant changes, whereas sedentary mice showed a sharp decline, which was closely associated with the worsening of vital status after IS (Chiu et al., 2012; Middleton et al., 2015; Grau-Pellicer et al., 2019). While there are limitations in predicting prognostic outcomes in this short-term model, early rapid recovery may suggest a better prognosis. Long-term outcome observations will be discussed in future experiments. Additionally, we acknowledge that this study did not account for aging as a biological variable. Given that aging is a major non-modifiable risk factor that worsens stroke severity and impairs recovery, future studies are warranted to examine whether the protective effects of EP on acute recovery are preserved across different age groups.

PTI surgery, while deviating slightly from the pathological mechanisms of natural cerebral ischemia, is minimally invasive and highly reproducible, enabling the induction of ischemia in specific areas (Uzdensky, 2018; Knezic et al., 2022). In our research, PTI mice exhibited increased levels of stroke-related markers such as S100B, NT-pro BNP, and GFAP, compared to the Con mice, which is typically observed during IS. Due to the neuroprotective effects of exercise on IS (Otsuka et al., 2016), we hypothesized lower levels of stroke markers in EP-PTI than in Sed-PTI mice. However, we did not observe statistically significant differences between Sed-PTI and EP-PTI mice in these three markers. One reason for this may be the time point of sacrifice, which corresponded to the early stage of IS. Although stroke-related behaviors typically recover within 3 days, there are cases where recovery may not occur even after 28 days (Han et al., 2023). Consistent with this, it may be too early to assess such biomarkers. Moreover, Otsuka et al., 2016 reported findings consistent with ours, indicating no significant differences in GFAP levels between sedentary and exercised mice 2 days after IS.

We evaluated blood–brain barrier (BBB) integrity through the expression levels of two key tight junction proteins, ZO-1 and occludin. Interestingly, ZO-1 expression was lower in EP-PTI than in both Con (p = 0.0072) and Sed-PTI (p = 0.0014) mice (Supplementary Figure S5, η^2^ = 0.71). While reduced ZO-1 expression is often interpreted as a marker of compromised BBB integrity, some reports suggest that ZO-1 overexpression can also increase the secretion of neutrophil chemoattractants, such as IL-8, GROα, and GM-CSF, thereby promoting neutrophil infiltration (Neyrinck-Leglantier et al., 2021). This heightened infiltration of neutrophils has been associated with increased neuroinflammation and exacerbation of stroke pathology. In the context of our findings, the lower ZO-1 levels observed in the EP-PTI group may be related to reduced neutrophil-associated chemotactic signaling, which aligns with our observations of decreased neutrophil migration (Figure 4B) and significantly downregulated expression of chemotactic markers such as fMLP and CXCR2 (Figures 3B,C). To further assess potential BBB disruption, we also examined occludin expression and found no significant differences among groups, suggesting that gross BBB permeability may not be markedly altered. Taken together, our results support the notion that the observed changes in ZO-1 may be more closely linked to neutrophil dynamics rather than structural BBB breakdown. Nonetheless, further validation using direct BBB permeability assays (e.g., Evans blue or IgG extravasation) will be necessary in future studies.

We propose neutrophils as the central mediator of IS (Vallés et al., 2017; Nayak et al., 2022; Zhao et al., 2023). IS can be ameliorated through the inhibiting or depletion of neutrophils, suggesting the independent role of neutrophils in IS pathology. The number of infiltrated and accumulated neutrophils may correlate with the severity of IS, emphasizing the importance of their migration capacity to circulate and arrive at the injury site during the early stage of IS. Furthermore, NETs have been identified as a key factor exacerbating IS (Kang et al., 2020; Zhou et al., 2020; Denorme et al., 2022; Zhao et al., 2023). Following the IS, circulating neutrophils increase at 1 day and infiltrate the infarct site of the brain, peaking at 24 h (Ruhnau et al., 2017; Cai et al., 2020; Mülling et al., 2021). In addition, NET formation in the cortex increases between 1 day and 3 days. Consequently, we conducted observations 2 days after PTI to verify the functions of neutrophils among groups, as the period between 1 day and 3 days emerges as a critical time frame for neutrophils in the context of IS.

Although exercise is known to reduce neutrophil migration (Gavrieli et al., 2008; Borges et al., 2018) and NET formation (Yazdani et al., 2021), its effects within the context of IS remain unclear. To this end, we assessed the effects of EP on neutrophil quantity, maturation, infiltration, and chemotactic signaling using flow cytometry, blood cell analysis, and RT-qPCR. (Figure 3; Supplementary Figures S2,S3). While total circulating myeloid cell counts remained unchanged under IS conditions, Ly6G expression in the infarcted cortex was significantly reduced following EP, suggesting a decrease in neutrophil infiltration into the brain. Moreover, EP notably suppressed the expression of the chemotactic mediators fMLP and CXCR2, both of which are known to enhance neutrophil migration and promote NET formation. This reduction in chemotactic signaling suggests that EP may limit the activation and inflammatory potential of infiltrating neutrophils.

After confirming the overall number and chemotactic potential of neutrophils through these experiments, we next investigated functional changes intrinsic to the neutrophils themselves. Our migration assay revealed that neutrophils from EP-PTI mice exhibited reduced migration capacity compared to those from Sed-PTI mice, indicating that EP attenuates their ability to reach and accumulate at the infarct site. Critically, we also observed that EP reduced NET levels in both plasma and brain tissue. NET levels in plasma were increased in Sed-PTI mice, while those in EP-PTI mice were significantly reduced, reaching levels comparable to Con. Additionally, NET formation in the brain was also significantly reduced in EP-PTI compared to Sed-PTI mice. While the assessment of NET levels in plasma provided an overview of the overall concentration in the body, NET evaluation in brain tissue revealed localized NET production by infiltrating neutrophils. Following EP, levels of NETs, which are elevated in IS patients and correlated with IS severity (Vallés et al., 2017), were reduced in both plasma and brain. This reduction in NETs may contribute to improved acute-phase recovery. Taken together, EP not only suppresses neutrophil chemotaxis and migratory capacity but also reduces NET formation, thereby contributing to improved acute-phase recovery following PTI. Although the need to utilize blood samples for both ELISA and neutrophil isolation resulted in reduced sample sizes for each experiment, this study effectively demonstrated the potential reduction in NET formation and migration following EP. Furthermore, by presenting the corresponding effect sizes, we were able to reinforce the reliability and robustness of these findings despite the limited sample numbers.

## 5 Conclusion

In conclusion, our results indicate the neuroprotective effects of EP during acute recovery in IS, demonstrating that EP-induced neutrophils may orchestrate critical mechanisms to alleviate IS. This is the first study to demonstrate that EP not only reduces neutrophil migration but also decreases the formation of NETs in both the circulation and the brain under IS conditions. These findings provide novel insights into how EP-induced neutrophils influence IS and highlight their potential relevance to diseases related to exercise-induced neutrophils.

## Data Availability

The raw data supporting the conclusions of this article will be made available by the authors, without undue reservation.

## Abbreviations

CitH3, citrullinated histone H3; Con, control; ELISA, enzyme-linked immunosorbent assay; EP, exercise preconditioning; EP-PTI, exercise-preconditioned-PTI; GFAP, glial fibrillary acidic protein; IF, immunofluorescence; (IS), ischemic stroke; NT-pro BNP, N-terminal pro-b-type natriuretic peptide; NETs, neutrophil extracellular traps; PTI, photothrombotic ischemic stroke; S100B, S100 calcium-binding protein B; Sed-PTI, sedentary-PTI.
