# Correction: Exercise preconditioning alleviates photothrombotic ischemic stroke in mice by orchestrating neutrophils

**DOI:** 10.3389/fphys.2025.1661262

**Published:** 2025-08-15

**Authors:** Tae Yeon Kim, Yun Seo Cho, Jae Yeon Park, Songwon Woo, Kihoon Yuk, Dong Heon Yi, In cheol Jeong, Jin Pyeong Jeon, Yoe-Sik Bae, Min-Chul Lee, Hyo Youl Moon

**Affiliations:** ^1^ Department of Physical Education, Seoul National University, Seoul, Republic of Korea; ^2^ School of Artificial Intelligence Convergence, Hallym University, Chuncheon, Republic of Korea; ^3^ Department of Population Health Science and Policy, Icahn School of Medicine at Mount Sinai, New York, NY, United States; ^4^ Department of Neurosurgery, Hallym University College of Medicine, Chuncheon, Republic of Korea; ^5^ Department of Biological Sciences, Sungkyunkwan University, Suwon, Republic of Korea; ^6^ Department of Sports Medicine, College of Health Science, CHA University, Pocheon, Republic of Korea; ^7^ Learning Sciences Research Institute, Seoul National University, Seoul, Republic of Korea; ^8^ Institute of Aging, Seoul National University, Seoul, Republic of Korea

**Keywords:** exercise preconditioning, neutrophil extracellular traps, migration capacity, photothrombotic ischemic stroke, voluntary exercise

In the published article, there was an error in affiliation(s) [1]. Instead of “[Exercise Biochemistry Lab, Department of Physical Education, Seoul National University, Seoul, Republic of Korea]”, it should be “[Department of Physical Education, Seoul National University, Seoul, Republic of Korea]”.

In the published article, there was an error in affiliation(s) [5]. Instead of “[Department of Biological Sciences, College of Natural Science Sungkyunkwan University, Jongno-gu, Republic of Korea]”, it should be “[Department of Biological Sciences, Sungkyunkwan University, Suwon, Republic of Korea]”.

The original article has been updated.

