# Supplementary figures and images for: Exercise preconditioning alleviates photothrombotic ischemic stroke in mice by orchestrating neutrophils
Source: Front Physiol. 2025 Jul 4;16:1580283. doi: 10.3389/fphys.2025.1580283 (PMC12273507; doi:10.3389/fphys.2025.1580283)

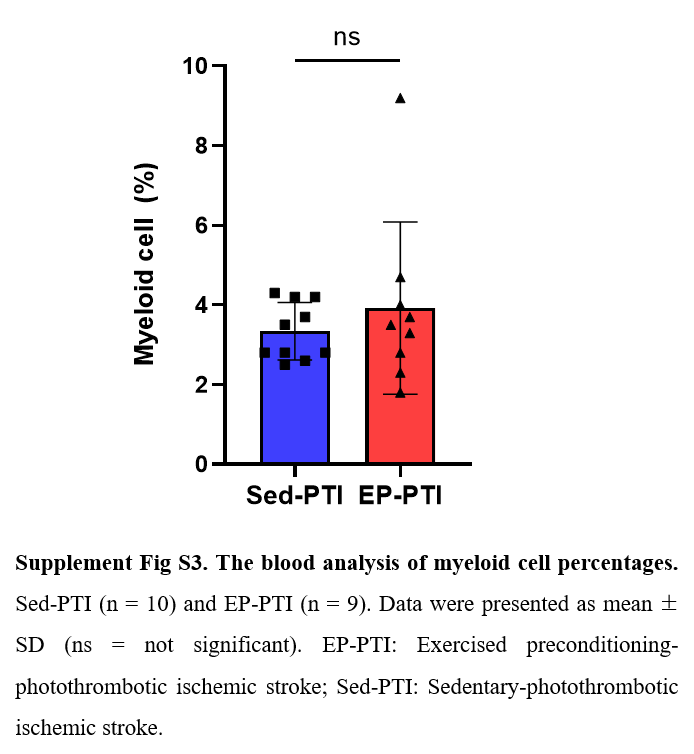

Supplement: Supplementary file 1 [file Image3.tif]

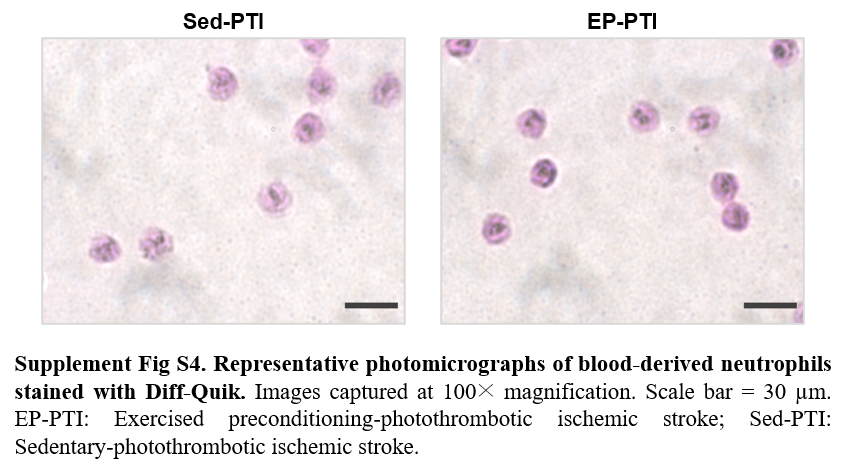

Supplement: Supplementary file 2 [file Image4.tif]

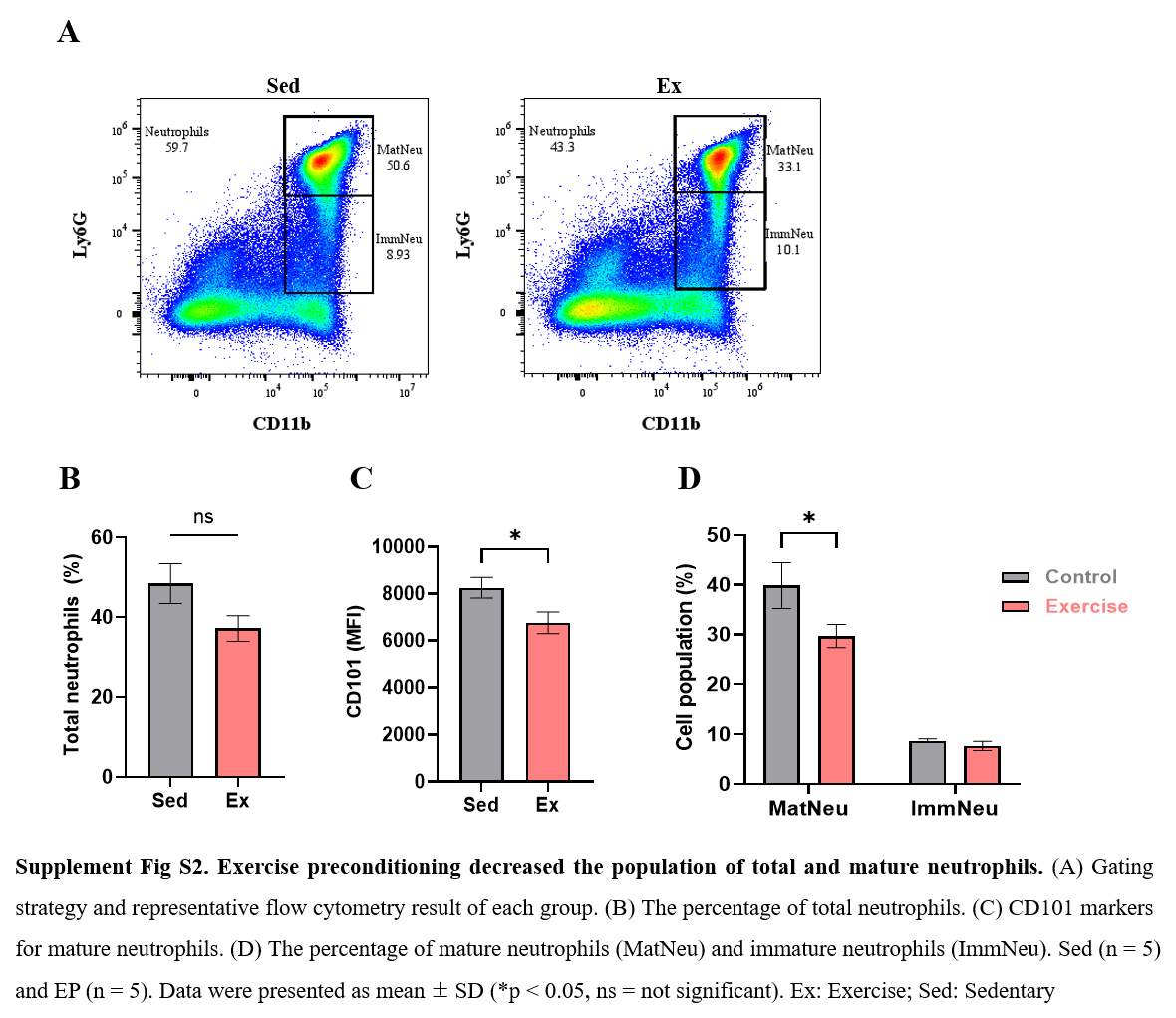

Supplement: Supplementary file 3 [file Image2.tif]

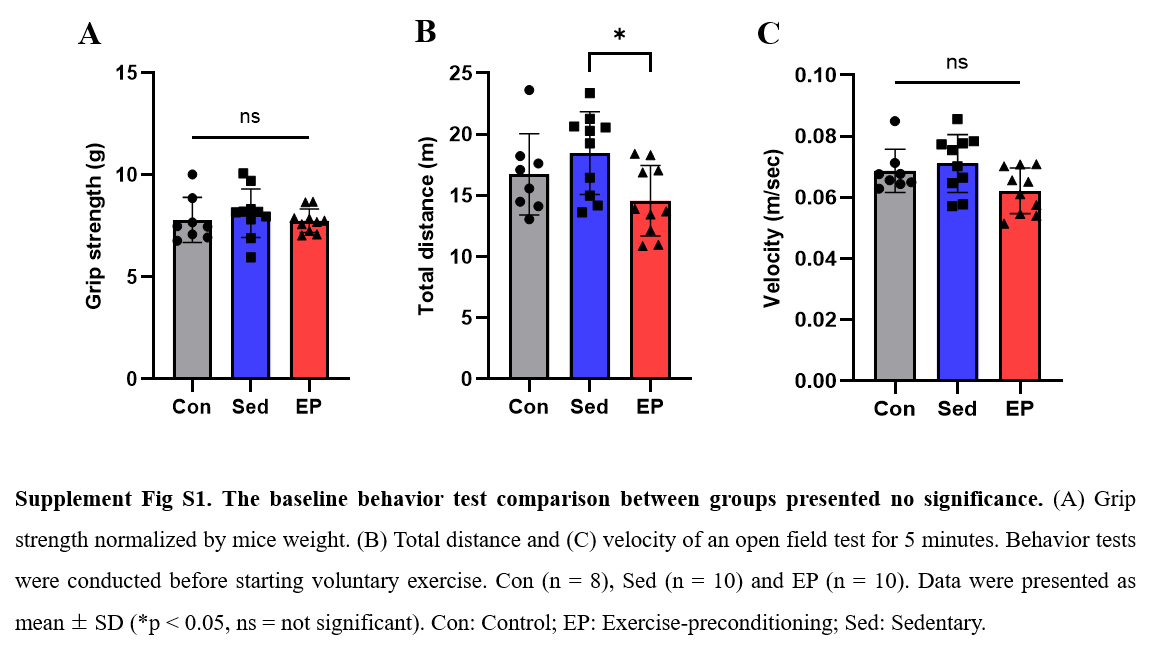

Supplement: Supplementary file 4 [file Image1.tif]

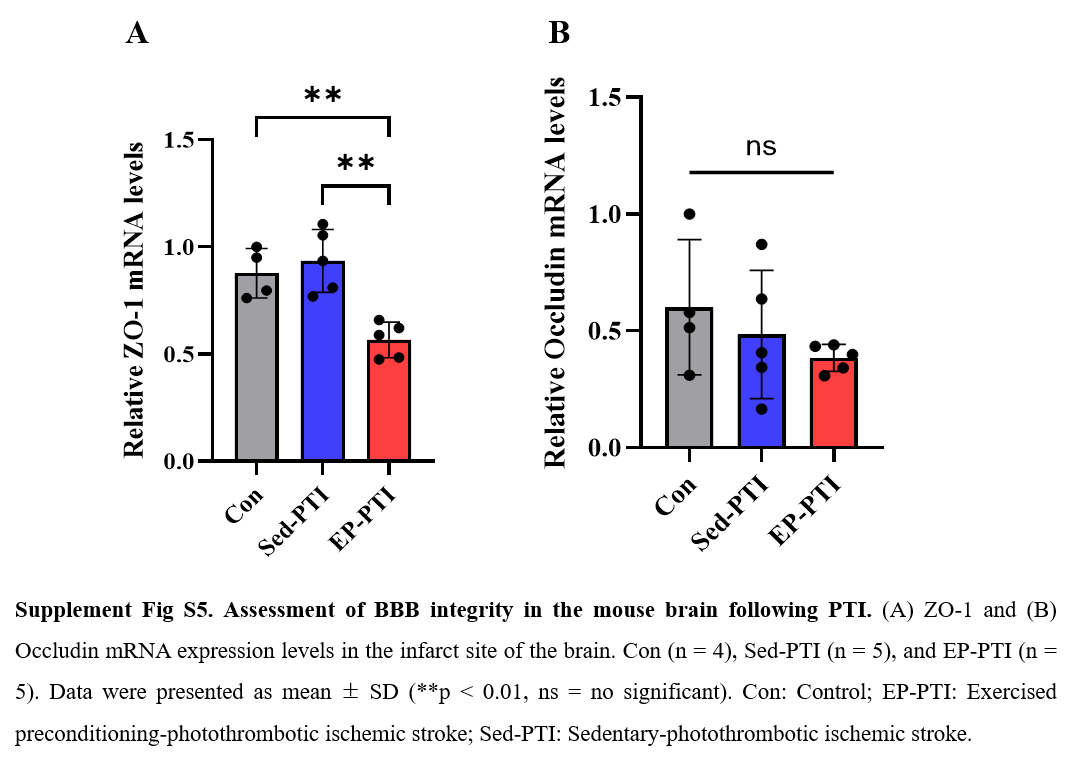

Supplement: Supplementary file 7 [file Image5.tif]
