# Supplementary material for: Exercise preconditioning alleviates photothrombotic ischemic stroke in mice by orchestrating neutrophils
Source: Front Physiol. 2025 Jul 4;16:1580283. doi: 10.3389/fphys.2025.1580283 (PMC12273507; doi:10.3389/fphys.2025.1580283)
Supplement: Supplementary file 5 [file Table2.docx]

Supplementary Material

# Supplementary Method

1.1 Flow cytometry

Neutrophils were isolated from the mice bone marrow (BM) by fluxing the tibia and femur with PBS. Red blood cells were lysed with an RBC lysis buffer (Gibco, Waltham, USA) for 10 minutes. BM cells were washed twice with PBS, and single-cell suspensions by RPMI1640 with 10% FBS were used for experiments.

Single cells were stained with zombi NIR fixable viability dye (1:1000, Biolegend) for 30 minutes at 4 °C. Subsequently, the cells were stained with anti-CD11b (eBioscience, California, USA), anti-Ly6G (eBioscience, California, USA), and anti-CD101 (eBioscience, California, USA) in PBS containing 0.5% of BSA with super bright complete staining buffer (Thermo Fisher Scientific, Waltham, USA) for 30 minutes at 4 °C. After washing twice with FACS buffer, cells were analyzed using Aurora flow cytometry (Cytek Bioscience, California, USA). Data were analyzed in FlowJo software (BD Bioscience, New Jersey, USA).

1.2 Blood cell analysis

2 days after PTI surgery, blood samples were collected using a heparin-containing tube. Whole blood samples were analyzed by an automated blood cell counter (Licare Biomedical Limited, Shenzhen, China).

# Supplementary Figures

**Supplementary Figure 1.** **Mouse body weight monitoring over 8 weeks.** Con (n = 8), Sed (n = 10) and Ex (n = 10). Statistical analysis was performed using two-way ANOVA with a Bonferroni post hoc test. Data were presented as mean ± SD (#p < 0.05, significant differences between Con and Ex. **p < 0.01, ***p < 0.001, significant differences between Sed and Ex. ns = not significant). Con: Control; Ex: Exercise-preconditioned; Sed: Sedentary.

**Supplementary Figure 2.** **The baseline behavior test comparison between groups.** (A) Grip strength normalized by mice weight. (B) Total distance and (C) velocity of an open field test for 5 minutes. Behavior tests were conducted before starting voluntary exercise. Con (n = 8), Sed (n = 10) and Ex (n = 10). Statistical analysis was performed using one-way ANOVA with a Bonferroni post hoc test. Data were presented as mean ± SD (*p < 0.05, ns = not significant). Con: Control; Ex: Exercise-preconditioned; Sed: Sedentary.

**Supplementary Figure 3.** **Exercise preconditioning decreased the population of total and mature neutrophils.** (A) Gating strategy and representative flow cytometry result of each group. (B) The percentage of total neutrophils. (C) CD101 markers for mature neutrophils. (D) The percentage of mature neutrophils (MatNeu) and immature neutrophils (ImmNeu). Sed (n = 5) and Ex (n = 5). Statistical analysis was performed using a student’s t-test. Data were presented as mean ± SD (*p < 0.05, ns = not significant). Ex: Exercise-preconditioned; Sed: Sedentary.

**Supplementary Figure 4.** **The blood analysis of myeloid cell percentages.** Sed-PTI (n = 10) and Ex-PTI (n = 9). Data were presented as mean ± SD (ns = not significant). Ex-PTI: Exercised-photothrombotic ischemic stroke; Sed-PTI: Sedentary-photothrombotic ischemic stroke.
